# Detection of Exostosin 1 in Lupus Nephritis: Prevalence, Clinico–Pathologic and Renal Outcome Correlations

**DOI:** 10.3390/diagnostics16111591

**Published:** 2026-05-23

**Authors:** Luiza Liza de Assis, Denise Maria Avancini Costa Malheiros, Dirce Maria Zanetta, Luis Yu

**Affiliations:** 1 Division of Nephrology, Hospital das Clinicas, University of Sao Paulo School of Medicine, Sao Paulo 05401-010, SP, Brazil; 2 Department of Pathology, University of Sao Paulo School of Medicine, Sao Paulo 05401-010, SP, Brazil; denise.mac.malheiros@gmail.com; 3 Department of Epidemiology, University of Sao Paulo School of Public Health, Sao Paulo 05401-010, SP, Brazil; dzanetta@usp.br

**Keywords:** membranous nephropathy, systemic lupus erythematosus, lupus nephritis, exostosin 1, kidney biopsy, prognosis

## Abstract

**Background/Objectives**: Lupus nephritis (LN) is one of the most severe complications of systemic lupus erythematosus (SLE); it is associated with increased morbidity and mortality, underscoring the need for new diagnostic markers and therapeutic strategies. In this context, the exostosin 1 (EXT1)/exostosin 2 (EXT2) heterodimer has emerged as a novel antigen in membranous nephropathy associated with SLE. This study evaluated EXT1 prevalence in renal biopsies from patients with lupus membranous nephropathy (LMN) and compared clinical, laboratory, and histopathological characteristics on diagnosis and renal outcomes. **Methods**: This retrospective study included 97 LMN patients whose renal biopsy underwent immunohistochemistry (IHC) for EXT1. EXT1-positive and EXT1-negative groups were compared using descriptive analyses and repeated measures models. **Results**: EXT1 positivity was observed in 35% of the cohort, and is more frequent in pure LMN (40%) than in cases with a proliferative component (32%). Regarding SLE diagnostic criteria, EXT1-positive patients showed a higher frequency of antiphospholipid antibodies, although data were available for only a subset of patients. This group also exhibited lower serum creatinine levels, but without statistical significance. EXT1-negative patients more frequently received cyclophosphamide as induction therapy (57.6% vs. 34.5%; *p* = 0.041). No differences in clinical outcomes were observed during follow-up. **Conclusions**: EXT1 prevalence was consistent with the literature, reinforcing the epidemiological reproducibility of this marker. EXT1-positive and EXT1-negative groups did not differ regarding clinical presentation, disease progression, and renal outcomes, heightening the need for prospective studies to further elucidate the diagnostic and prognostic role of EXT1 in LMN.

## 1. Introduction

Membranous nephropathy (MN) is an autoimmune glomerular disease caused by deposition of antigen–antibody complexes in the subepithelial region of the glomerular basement membrane (GBM). It is the leading cause of nephrotic syndrome in adults [1,2,3], accounting for 20 to 37% of cases in non-diabetic patients [4], with an incidence of 2 to 4 cases per million inhabitants [5]. It predominantly affects males (2:1) with a mean age of presentation in the fifth decade of life, although it can affect individuals from childhood to advanced age [6]. MN is classified as primary (70–80% of cases) or secondary, when resulting from other conditions (autoimmune diseases, malignancies, infections, or drugs) [1,2,6,7]. However, recent advances in target antigen identification have challenged this dichotomized classification [5,8,9].

In 2009, Beck et al. identified the main podocyte antigen in primary MN in adults: the M-type phospholipase A2 receptor (PLA2R) [10]. A second podocyte antigen was identified in 2014—thrombospondin type-1 domain-containing 7A (THSD7A); it is less frequent and more associated with malignancies [11]. These findings demonstrated that most MN cases harbored antibodies against podocyte antigens, with PLA2R and THSD7A as the main representatives [1,3].

Despite these advances, a subset of patients with primary MN remained seronegative for both PLA2R and THSD7A, and many of these cases shared a common feature—strong association with autoimmune diseases, particularly systemic lupus erythematosus (SLE)—suggesting the existence of yet unidentified target antigens. In 2019, Sethi et al., through laser microdissection and mass spectrometry, identified the proteins EXT1 and EXT2 in this context [3]. Patients positive for these antigens exhibited clinical and laboratory features consistent with autoimmune diseases and renal biopsies revealed a histological pattern characteristic of secondary forms of the disease: C1q deposits in more than 70% of cases, a full-house immunofluorescence pattern in 80%, a predominance of IgG1 immunoglobulins, and frequent subendothelial and mesangial deposits [5].

Based on these findings, studies began to specifically investigate patients with lupus membranous nephropathy (LMN). EXT positivity was reported in 26 to 38.4% of cases, with higher rates among patients with pure class V [7,12,13,14,15,16]. Several studies suggest that positive patients presented lower creatinine levels [7,14,15,17], lower activity [7,14,15], and chronicity indices on renal biopsy [7,12,14,15,17], but a higher frequency of nephrotic-range proteinuria on diagnosis [7,12,,18]. However, these findings are not consistent across the literature; some studies found no differences in clinical presentation [16,19], while others observed no higher proteinuria levels in EXT1/EXT2-positive patients [14,15,16,17,19].

Similar discrepancies exist regarding outcomes: some evidence suggests that EXT1/EXT2-positive patients achieve better response rates [12,17,18] and lower progression to chronic kidney disease (CKD) [7,17,18], while other studies found no differences between groups [14,15,16].

Another important question remains unresolved: the observation of granular EXT1/EXT2 positivity mirroring immunoglobulin deposits raises the hypothesis that exostosins may serve as target antigens in lupus membranous nephropathy. However, to date, no circulating antibodies against EXT1/EXT2 have been identified [12]. Nevertheless, it seems unlikely that exostosin is secreted by podocytes in response to immune complex deposition, given that its expression appears to be specific to lupus membranous nephropathy [3,12]. These gaps underscore the need for further studies.

Finally, the expression of EXT in LMN has been evaluated in few populations, with no representation of Brazilian patients. Given the high prevalence of SLE in Brazil [20] and the remarkable ethnic diversity, investigating EXT expression in this population is highly relevant. Therefore, the objectives of the present study were 1. to determine the prevalence of EXT1 positivity in patients with pure LMN (class V) and associated with proliferative component (class III/IV+V); 2. to compare clinical and histopathological characteristics between EXT1-positive and EXT1-negative patients on diagnosis; and 3. to evaluate clinical outcomes, with the primary composite endpoint being a sustained decline in estimated glomerular filtration rate (eGFR) ≥40% from baseline or progression to end-stage kidney disease (ESKD), and secondary endpoints including complete response, primary efficacy renal response, partial response, and no kidney response to treatment.

## 2. Materials and Methods

### 2.1. Study Design and Population

This is a retrospective observational cohort study conducted using data collected from January 2014 to December 2022. Patients aged 18 years or older with a diagnosis of lupus nephritis (LN) class V or III/IV + V, according to the ISN/RPS classification [21], confirmed by renal biopsy performed at the Pathology Department of Hospital das Clinicas, Sao Paulo, Brazil, were eligible for inclusion. Patients without available demographic, clinical, or laboratory data in the electronic medical records, as well as those without sufficient histological material for immunohistochemical (IHC) analysis, were excluded.

Controls were selected by convenience sampling and included six cases of proliferative lupus nephritis without a membranous component, seven cases of primary membranous nephropathy with positive serum anti-PLA2R, and one case of membranous nephropathy (MN) with positive tissue PLA2R.

### 2.2. Clinical and Laboratory Data Collection

The electronic medical records of all included patients were reviewed. Demographic, clinical, and laboratory data were collected at the time of diagnosis, along with follow-up information obtained throughout the observation period, from January 2014 to December 2022.

### 2.3. Immunohistochemical Analysis

Histological sections of 3–4 micrometers in thickness were obtained from stored paraffin-embedded tissue blocks. IHQ for EXT1 was performed using a rabbit polyclonal anti-exostosin-1 antibody (Thermo Scientific, Itapevi, SP, Brazil, #PA5-60699) at a dilution of 1:100. Technical details of the procedure are provided in Supplementary Materials Section S1.

Slides were examined under light microscopy by a nephropathologist blinded to patients’ clinical data. Inter-observer reliability assessment was not performed. EXT1 positivity was assessed across the glomerular, tubulointerstitial, and vascular compartments. In glomeruli, deposits were identified along the capillary walls and graded according to their distribution pattern (focal or diffuse; segmental or global) and staining intensity, classified into four grades: 0 (absent), 1+ (weak), 2+ (moderate), and 3+ (strong). A case was classified as EXT1-positive when staining intensity was ≥1+, with a granular distribution along the glomerular capillary wall. When present, positivity was observed in 100% of the glomeruli evaluated in the sample. Non-specific staining in tubules and podocytes, as well as segmental and focal positivity with weak intensity, were disregarded.

The decision to evaluate EXT1 alone was based on evidence from the recent literature, which indicates that independent staining of EXT1 or EXT2 in the absence of the other has not been described in the context of membranous nephropathy [5,9], and the staining intensity of EXT1 has been reported as a little brighter when compared with EXT2 in the positive cases [7]. The antibody used was the same as that employed in the initial studies describing this finding, ensuring methodological comparability.

### 2.4. Statistical Analysis

Descriptive analysis of quantitative variables was performed by calculating the mean, standard deviation, minimum and maximum values, median, quartiles, and interquartile range. Categorical variables were presented as absolute frequencies and percentages. Analyses were stratified by time point (presentation and follow-up) and by pre-defined subgroups (mixed class, class V, and patients without induction therapy).

The normality of quantitative variables was assessed using the Shapiro–Wilk test. For normally distributed variables, between-group comparisons were performed using Student’s *t*-test; for non-normally distributed variables, the non-parametric median test was applied. Associations between categorical variables were evaluated using the chi-square test.

Renal survival was assessed using Kaplan–Meier curves and log-rank tests for ESKD, renal replacement therapy (RRT), and sustained eGFR decline ≥40% from baseline.

Repeated measures models were fitted to evaluate the effects of group, time, and group-by-time interaction on quantitative variables. For normally or approximately symmetrically distributed variables, repeated measures ANOVA was used, followed by Tukey’s multiple comparison test. For markedly skewed variables, a gamma distribution model was fitted, with multiple comparisons performed using the Wald test. For binary variables, repeated measures logistic regression models were applied. To explore distinct sources of confounding, two models were constructed separately for each outcome: the first adjusted for clinical and laboratory variables (activity and chronicity indices and C3 and C4 levels) and the second adjusted for the initial induction therapy used.

The primary outcome was assessed by the change in estimated glomerular filtration rate (eGFR) over the follow-up period, calculated using the 2021 CKD-EPI equation (Chronic Kidney Disease Epidemiology Collaboration). End-stage kidney disease (ESKD) was defined as an eGFR below 15 mL/min/1.73 m^2^ or dependence on renal replacement therapy for more than three months [22]. Secondary outcomes were analyzed according to the response criteria established by KDIGO 2024 [23].

A significance level of 5% (*p* < 0.05) was adopted for all analyses. All statistical analyses were performed using SAS for Windows, version 9.4.

## 3. Results

**Figure 1:**
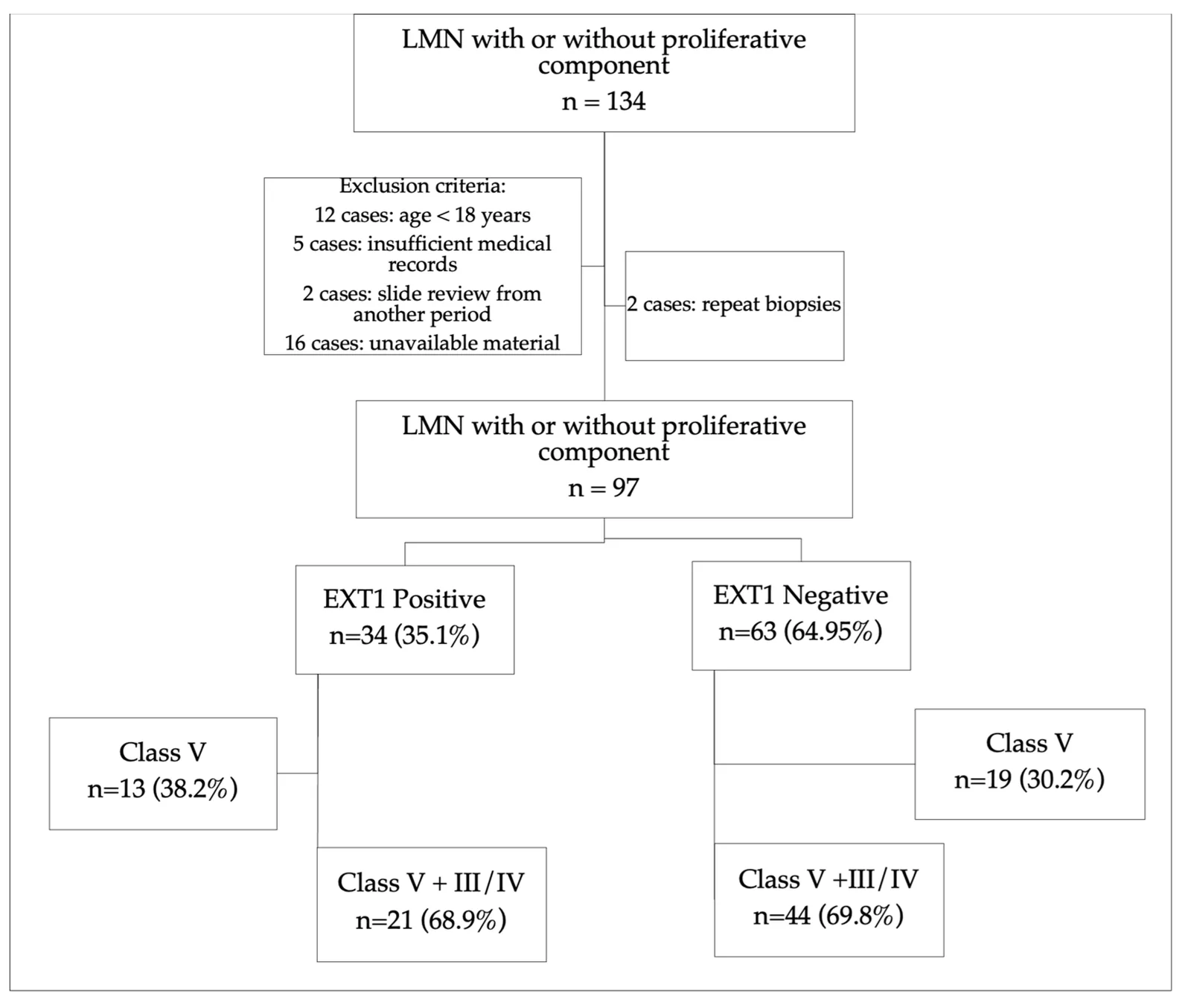
Study cohort flowchart. LMN: Lupus Membranous Nephropathy.

A total of 97 patients were included in the study. Data on the timing of SLE diagnosis were available for 94 patients: 75.5% had a prior diagnosis of SLE, while 24.5% were diagnosed concomitantly with lupus nephritis. EXT1 positivity was identified in 35.1% of kidney biopsies. None of the control cases showed positive staining. Figure 1 illustrates the flowchart of cases selection.

Most patients were female (84/86.6%). Regarding race/ethnicity, among the 96 patients with available data, 62 (64.6%) were White, 33 (34.4%) were Black or Mixed-race and 1 (1.0%) was Asian. The median age was 35 years (IQR: 28–42). At the time of renal biopsy, 33.7% of patients with available information had already initiated induction therapy with cyclophosphamide, mycophenolate, or rituximab, with a median prior treatment duration of 4 months. Additionally, 38.0% were on maintenance therapy with mycophenolate, azathioprine, or another immunosuppressive regimen at the time of relapse requiring renal biopsy; however, data on the duration of prior maintenance therapy were not systematically available in our cohort and could not be included in the analysis. The median eGFR was 102 mL/min/1.73 m^2^. The median proteinuria on diagnosis was 1.77 g; 24-h urine protein was used whenever available; otherwise, the urine protein-to-creatinine ratio (UPCR) was considered. Regarding the histological pattern, 67% of patients presented an associated proliferative component. Table 1 summarizes the clinical, laboratory, and histological characteristics at the time of renal biopsy.

### 3.1. EXT1-Positive Patients

IHC analysis disclosed 35.1% of patients positive for EXT1. None of the control cases showed positive staining. The median age of EXT1-positive patients was 37 years and 79.4% were female.

Among EXT1-positive patients, 68.8% had a prior SLE diagnosis. At the time of renal biopsy, 28.1% had already initiated induction therapy with cyclophosphamide, mycophenolate, or rituximab, with a median prior treatment duration of 5 months, and 34.4% experienced a relapse during maintenance therapy with azathioprine, mycophenolate, or another immunosuppressive regimen, which prompted the biopsy. Regarding SLE diagnostic criteria, although these data were available for only 59 patients (14 EXT1-positive patients (41%) and 24 EXT1-negative patients (38%); *p* = 0.766), EXT1-positive patients had a significantly higher frequency of positive antiphospholipid antibodies (40%), with no correlation with thrombotic microangiopathy at the time of renal biopsy.

No statistically significant differences were observed between EXT1-positive and EXT1-negative groups for any clinical or laboratory parameters evaluated on presentation, although lower creatinine, hematuria, and proteinuria were observed in the EXT1-positive group. No differences were also observed in nephrotic-range proteinuria, serum albumin, or serum cholesterol on presentation.

Concerning complement levels, 38.2% of EXT1-positive patients presented both C3 and C4 consumption. Anti-dsDNA was positive in 71.0% of the patients. None of these variables differed significantly compared to the EXT1-negative group.

Analyzing histopathological findings, 61.8% EXT1-positive patients had an associated proliferative component. Crescents were observed in 26.5%, and C1q deposits were found in 87.9% of the biopsies. Activity and chronicity indices were lower in EXT1-positive patients, although without statistical significance. Clinical, laboratory, and pathological characteristics comparing EXT1-positive and EXT1-negative groups are presented in Table 2 and Table 3. A sensitivity analysis restricted to patients who had not yet initiated induction therapy at the time of biopsy was performed, yielding no statistically significant differences, and it is presented in Supplementary Table S1.

**Figure 2:**
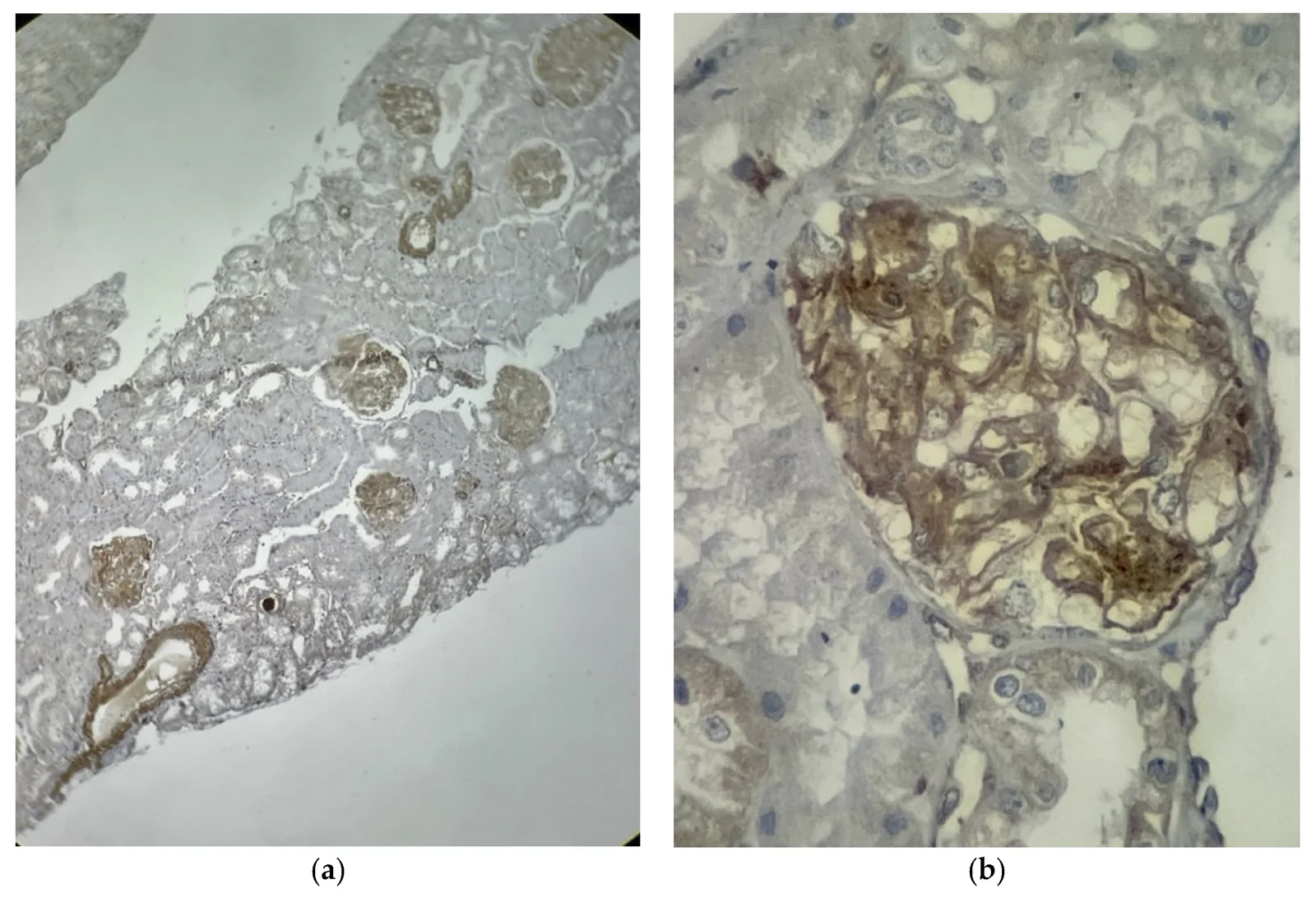
Immunohistochemistry of patient with positive exostosin 1: (**a**) Patient 65, 400×; (**b**) Higher, 100× magnification.

**Figure 3:**
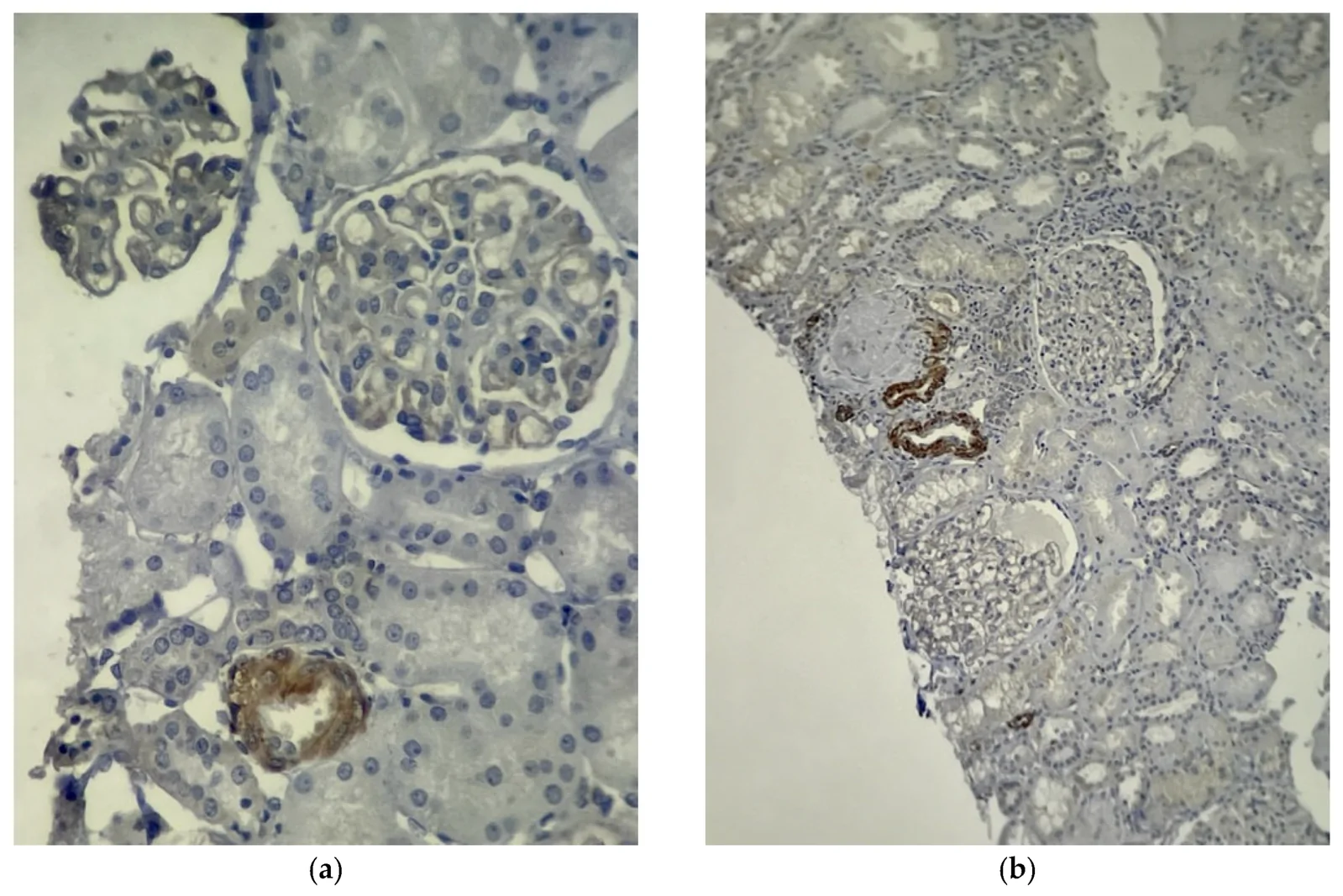
Immunohistochemistry of patients with negative exostosin 1: (**a**) Patient 3; (**b**) Patient 4.

IHC staining images are shown in Figure 2 and Figure 3.

### 3.2. Clinical Follow-Up of EXT1 Positive and Negative LMN Patients

Out of 97 patients, 88 had available data on initial therapy—induction followed by maintenance treatment. Among EXT1-negative patients, induction therapy with cyclophosphamide was used more frequently (57.6% vs. 34.5%; *p* = 0.041), despite a similar frequency of proliferative findings between groups (72.8% vs. 65.5%; *p* = 0.476). Conversely, among EXT1-positive patients, mycophenolate was more frequently chosen as induction therapy (44.8% vs. 22.0%; *p* = 0.027).

#### Clinical Follow-Up of LMN Patients With or Without Proliferative Component

At one year follow-up, complete data were available for 88 patients, decreasing to 84 after two years, 72 after three years, and 62 at the end of four years of follow-up. This decrease was primarily due to censoring by study follow-up time. The median follow-up time did not differ significantly between EXT1-positive and EXT1-negative patients [48.0 (IQR 33.5–84.5) vs. 48.0 (IQR 34.0–61.0) months, respectively; *p* = 0.497]. Supplementary Table S2 presents a detailed overview of sample size evolution and the specific reasons for each exclusion. The proportion of patients with proliferative features on kidney biopsy did not differ between groups over time, nor did the need for re-induction or maintenance regimen used. No statistically significant difference was observed in the primary outcome—sustained eGFR reduction ≥40% from baseline or progression to ESKD—or in the secondary outcomes, including complete response, primary effective renal response, partial response, and no response to treatment. No difference in mortality was observed between groups. After the first year, EXT1-positive patients more frequently showed C3 and C4 consumption; however, this difference was not sustained over time. Clinical characteristics and outcomes are presented in Table 4, Table 5, Table 6 and Table 7.

**Figure 4:**
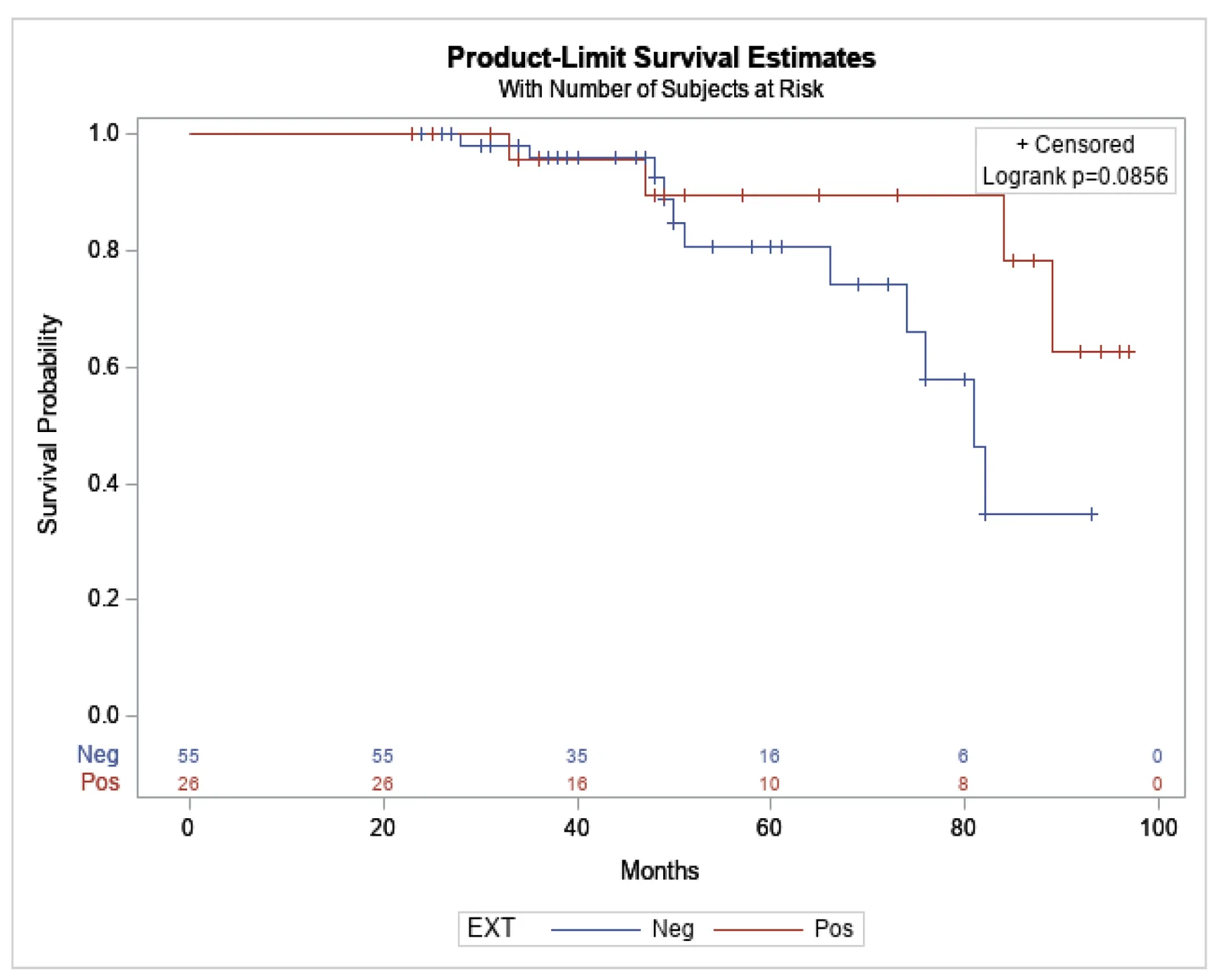
Kaplan–Meier survival curves comparing eGFR decline ≥40% in EXT1-positive and EXT1-negative groups.

No significant differences were observed between groups in renal survival free of ESKD (*p* = 0.275), free of RRT (*p* = 0.214), or free of eGFR decline ≥40% (*p* = 0.080). Notably, regarding this latter outcome, a greater renal function loss was observed in the EXT1-negative group, although without reaching statistical difference. Kaplan–Meier survival curves comparing eGFR decline ≥40% in EXT1-positive and EXT1-negative groups are presented in Figure 4. Survival curves for ESKD and RRT have been included as Supplementary Figures S1 and S2, respectively.

Two separate repeated measures models were constructed to evaluate creatinine and proteinuria levels between groups over follow-up: the first adjusted for clinical and laboratory variables (activity and chronicity indices and C3 and C4 levels) and the second adjusted for the initial induction therapy used. The models were fitted based on the data available at each time point, with sample sizes varying according to covariate availability: 86 patients at year one, 83 at year two, 70 at year three, and 50 at year four of follow-up. After adjustment, no statistically significant differences were observed in creatinine between EXT1-positive and EXT1-negative groups in either the first (*p* = 0.358) or the second model (*p* = 0.809), as shown in Figure S3. Similarly, no significant differences were observed in proteinuria levels in the first (*p* = 0.626) or the second model (*p* = 0.734), as shown in Figure S4.

The same approach was applied to evaluate treatment responses over time, with the addition of creatinine and proteinuria as covariates in the first model. Sample sizes varied according to covariate availability: 86 patients at year one, 82 at year two, 68 at year three, and 51 at year four of follow-up. In the first model, adjusted for clinical and laboratory variables (Figure S5), no statistically significant differences were observed between groups regarding complete response (*p* = 0.974), primary effective renal response (*p* = 0.592), partial response (*p* = 0.401), or no response (*p* = 0.435). The second model, adjusted for induction therapy (Figure S6), confirmed the absence of differences: complete response (*p* = 0.754), primary effective renal response (*p* = 0.473), partial response (*p* = 0.312), and no response (*p* = 0.560).

The models were applied to six-year follow-up data, with 28 patients available at year five and 22 at year six. In the model adjusted for clinical and laboratory variables, no statistically significant differences were observed between groups in creatinine evolution (*p* = 0.429), proteinuria levels (*p* = 0.768), complete response (*p* = 0.681), primary effective renal response (*p* = 0.306), partial response (*p* = 0.906), or no response (*p* = 0.972). The model adjusted for initial induction therapy confirmed the absence of significant differences for the same outcomes: creatinine (*p* = 0.780), proteinuria (*p* = 0.903), complete response (*p* = 0.452), primary effective renal response (*p* = 0.297), partial response (*p* = 0.905), and no response (*p* = 0.957).

### 3.3. Follow-Up According to Histological Classification

Regarding patients with available follow-up data, 13/34 (38.2%) EXT1-positive patients and 19/63 (30.2%) EXT1-negative patients had pure class V lupus nephritis. After one year of follow-up, 10/10 (100%) EXT1-positive patients maintained positive anti-dsDNA antibodies, compared to 7/15 (46.7%) EXT1-negative patients, (*p* = 0.007). However, over the course of follow-up, the frequency of positive anti-dsDNA no longer differed between groups. Similarly, no differences were observed between groups in the remaining characteristics.

Finally, when analyzing only patients with mixed-class LN, 21/34 (61.8%) were EXT1-positive and 44/63 (69.8%) were EXT1-negative. After one year of follow-up, in contrast to isolated class V LN, 5/15 (33.3%), EXT1-positive patients maintained positive anti-dsDNA antibodies, compared to 26/40 (65.0%) EXT1-negative patients (*p* = 0.003). As observed in the class V analysis, this difference was no longer detected in subsequent years. No statistically significant difference was observed in the primary outcome. At the end of 3 years of follow-up, EXT1-negative patients showed higher rates of complete and effective responses (*p* = 0.026), a difference that was not sustained after four years of follow-up.

## 4. Discussion

EXT1 and EXT2 were recently identified antigens associated with secondary membranous nephropathy (MN) in patients with autoimmune diseases, particularly SLE [5]. The exostosin family comprises glycosyltransferases responsible for heparan sulfate (HS) biosynthesis [24]. To understand their relevance in the glomerular context, it is essential to highlight the structure of the glomerular filtration barrier, composed of endothelial cells and podocytes. Between these two cell layers lies the glomerular basement membrane (GBM) [25], a highly specialized matrix in which HS is a central component [5]. Furthermore, the endothelial glycocalyx also relies on HS as its main glycosaminoglycan [26,27]. Another key role of HS is its contribution to the density of negative charges and, consequently, to charge-selective permeability [25].

In the present study, consistent with previous publications [7,12,15,17], EXT1 positivity was observed in 35% of cases, more frequent in patients with class V LN (40%) than in those with an associated proliferative component (32%). None of the control cases showed positive staining. This finding reinforces the epidemiological consistency of EXT1 across different populations and, considering the high prevalence of SLE in Brazil and the remarkable ethnic diversity of this population, these results highlight its relevance.

When evaluating the clinical presentation of patients in our cohort, EXT1-positive individuals showed a higher frequency of antiphospholipid antibodies among SLE diagnostic criteria (40% vs. 15.4%; *p* = 0.035). The proportion of missing data did not differ significantly between groups, suggesting that missingness was random and not systematically associated with either group. Nevertheless, the limited sample with available data reduces the statistical power of this comparison, and confirmation in larger, prospective cohorts is warranted.

Similarly to what is described in the literature [7,12,14,15,17], EXT1-positive patients showed lower creatinine levels and greater eGFR at presentation, although without statistical significance, which may be partially attributed to the sample size of the present study. Furthermore, the fact that a significant proportion of patients were already on immunosuppressive therapy at the time of biopsy may have influenced these findings, representing an additional confounding factor in the interpretation of baseline clinical characteristics. To minimize this effect, a sensitivity analysis was performed, and results remained consistent.

Regarding proteinuria levels, the heterogeneity across studies reinforces that the relationship between EXT1 positivity and proteinuria severity remains uncertain. While Ravindran et al. and other publications have reported higher proteinuria levels on diagnosis among EXT1-positive patients [7,12,18,28], other studies have not confirmed these findings [14,15,16,17,19]. In our cohort, the opposite pattern was observed: EXT1-negative patients showed higher proteinuria levels compared to EXT1-positive patients, although without statistical significance. This finding may be partially explained by the proportion of patients already on induction or maintenance therapy at the time of renal biopsy. The absence of electron microscopy data in our cohort further limits the interpretation of this finding, as podocyte ultrastructural assessment could provide additional insight into the relationship between EXT1 expression and proteinuria. Furthermore, based on the hypothesis that EXT1/EXT2-positive patients exhibit greater secretion of these glycosyltransferases into the GBM, resulting in increased HS synthesis, it is plausible that EXT1-negative individuals have reduced production of this glycosaminoglycan and HS loss has been implicated in the development of proteinuria across various glomerulopathies [7,29,30,31,32,33,34].

Among the histopathological findings, no significant differences were observed between EXT1-positive and EXT1-negative groups regarding the presence of an associated proliferative component, crescents, C1q deposits, or activity and chronicity indices.

Notably, despite similar presentation, the choice of induction therapy differed between groups, with cyclophosphamide being used more frequently among EXT1-negative patients and mycophenolate being more commonly chosen among EXT1-positive patients, despite a similar frequency of proliferative findings between groups. This pattern may support the hypothesis that EXT1-negative patients had a more severe initial presentation, given that in clinical scenarios of rapid renal function deterioration or marked histological activity, many clinicians prefer cyclophosphamide-based regimens.

Considering patients outcomes, the literature is controversial: some publications report an association between EXT1 positivity and higher response rates and lower progression to ESKD [7,8,12,17,18], while others found no significant differences between cohorts [13,14,15,16]. Several hypotheses have been proposed to explain the potential protective role of exostosin proteins: EXT1/EXT2-positive patients may exhibit increased secretion of the catalytic domain of these glycosyltransferases into the GBM, resulting in greater HS synthesis, which may directly protect against inflammatory injury, facilitating the clearance of extracellular proteins and pro-inflammatory factors [7], and modulate local complement activation via factor H [29] and promote adaptive immune responses and the maintenance of immunological self-tolerance [24]. Finally, HS has also been implicated in the development of renal fibrosis and CKD, through regulation of endothelial growth factors and cytokines function; thus, HS may contribute to the prevention of proteolysis, inflammation, and activation of pro-fibrotic pathways [30].

In the present study, no statistically significant differences were observed in the primary outcome—progression to ESKD or sustained eGFR reduction ≥40% from baseline, as illustrated by the Kaplan–Meier curves. Of note, regarding this outcome, a greater loss was observed in the EXT1-negative group. Furthermore, no differences were observed in the secondary outcomes. No difference in mortality was also observed between groups. However, most EXT1-negative patients received cyclophosphamide, while EXT1-positive patients received mycophenolate as induction therapy. To explore the potential confounding effects of induction therapy, the mixed models were additionally adjusted for the therapeutic regimen used. Even so, no significant differences between groups were observed.

Furthermore, after the first year of follow-up, EXT1-positive patients showed C3 consumption (30.8% vs. 12.1%; *p* = 0.030) and C4 consumption (30.8% vs. 10.3%; *p* = 0.020), more frequently. However, this difference was not sustained over time. This finding may reflect a distinct immunological phenotype in this subgroup. Similarly, the mixed models were adjusted for complement consumption, with no statistically significant differences observed between groups.

Analyzing the subgroups according to histological classification, after one year of follow-up, in the class V LN subgroup, 100% of EXT1-positive patients maintained positive anti-dsDNA antibodies, compared to 46.7% of EXT1-negative patients (*p* = 0.007); however, over the course of follow-up, the frequency of positive anti-dsDNA no longer differed between groups, and no significant difference was observed in the primary outcome. Conversely, in the mixed-class subgroup, 33.3% of EXT1-positive patients maintained positive anti-dsDNA antibodies, compared to 65.0% of EXT1-negative patients (*p* = 0.003), after one year, and over the course of follow-up, the frequency of positive anti-dsDNA no longer differed between groups. When analyzing only mixed-class LN patients, EXT1-negative patients showed higher rates of complete and effective responses at three years of follow-up (*p* = 0.026). This finding, however, derives from a subgroup with a limited number of patients, which restricts statistical power. Additionally, the difference was not sustained after four years of follow-up. Therefore, the lack of temporal consistency and the divergence from data reported in the literature suggest that this result likely reflects a sample size limitation.

Although serum anti-EXT1/EXT2 assays are not yet widely available in Brazil, EXT1 immunohistochemistry represents a feasible and informative tool that can be incorporated into the routine evaluation of renal biopsies in specialized centers, contributing to the diagnostic workup of membranous nephropathy and potentially informing prognostic assessment.

Finally, certain characteristics of the study design must be considered when interpreting the results. As a retrospective investigation, the analysis depends on the quality and completeness of available records, which may introduce biases inherent to this methodology. The cohort, although representative of the studied context, has a moderate sample size, and follow-up time was limited by censoring at the end of the study period, which may restrict both statistical power and the detection of differences in long-term outcomes. Consequently, the risk of type II error is substantial, and results should be interpreted with appropriate caution. Additionally, electron microscopy data were not available for this cohort, which precluded the assessment of podocyte effacement and its potential relationship with EXT1 expression and proteinuria. Inter-observer reliability assessment was not performed, which is acknowledged as an additional limitation of the study. Furthermore, the higher frequency of antiphospholipid antibodies observed in EXT1-positive patients should be interpreted as a preliminary finding, given that this information was unavailable for approximately 40% of patients in both groups. Thus, results confirmation in larger, prospective cohorts with complete data availability is warranted. Another relevant point is that a significant proportion of patients were already on treatment at the time of biopsy, a condition that may have influenced the evaluated outcomes. Specifically, 33.7% of patients were receiving induction therapy and 38% were on maintenance immunosuppression at the time of biopsy. The heterogeneity of immunosuppressive regimens used—including different agents, dosing strategies, and treatment durations prior to biopsy—represents a meaningful source of confounding that could have differentially affected histological findings and subsequent clinical outcomes across groups. Although adjusted analyses and sensitivity analyses were performed, residual confounding related to prior treatment cannot be fully excluded. It should be noted, however, that this study addresses a specific and relatively rare subtype of lupus nephritis, and all eligible cases identified over a six-year period were included in the analysis, which underscores its exploratory value despite the limitations of the study.

## 5. Conclusions

EXT1 positivity prevalence in our cohort was consistent with those reported in the literature, reinforcing the epidemiological reproducibility of this marker in the Brazilian population with remarkable ethnic diversity. EXT1-positive and EXT1-negative groups did not differ regarding clinical presentation, disease progression, and renal outcomes. EXT1-negative patients were more frequently treated with cyclophosphamide, which may suggest a more severe initial phenotype in this group. Prospective studies with standardized treatment protocols and adequate sample sizes, integrating diverse populations, are needed to elucidate the diagnostic and prognostic role of EXT1 in LMN.

## Figures and Tables

**Table 1:** Characteristics of the overall cohort at the time of renal biopsy.

Characteristics	Results
Prior SLE diagnosis (n = 94)	71/94 (75.5%)
Positive ANA (n = 67)	63/67 (94.0%)
Induction therapy (n = 92)	31/92 (33.7%)
Prior induction treatment duration (months)	4 (IQR: 2,5–6)
Cyclophosphamide	20/92 (21.0%)
Mycophenolate	11/92 (11.96%)
Rituximab	1/92 (1.0%)
Maintenance therapy (n = 92)	35/92 (38.0%)
Azathioprine	12/92 (13.0%)
Mycophenolate	19/92 (20.6%)
Other	4/92 (4.3%)
Hydroxychloroquine (n = 92)	49/92 (53.2%)
Corticosteroids (n = 92)	67/92 (72.8%)
RAAS blockers (n = 92)	56/92 (60.8%)
Creatinine (mg/dL) (n = 97)	0.82 (IQR: 0.64–1.32)
CKD-EPI 2021 eGFR (mL/min/1.73 m^2^) (n = 97)	102 (IQR: 64–120)
Urine red blood cells (cells/field) (n = 97)	4 (IQR:1–14)
Proteinuria (g) (n = 96)	1.77 (IQR 0.73–3,81)
Nephrotic-range proteinuria (n = 96)	26 (27.0%)
Serum albumin (g/dL) (n = 97)	3.3 (IQR: 2.6–3.7)
Serum total cholesterol (mg/dL) (n = 97)	230 (IQR: 190–265)
C3 consumption (n = 97)	32/97 (32.9%)
C4 consumption (n = 97)	26/97 (26.8%)
Anti-dsDNA positive (n = 91)	64/91 (69.57%)
Lupus membranous nephritis with proliferative component (n = 97)	65/97 (67.0%)
Crescents on renal biopsy (n = 97)	25/97 (25.7%)
C1q deposits (n = 93) =	82/93 (88.1%)
Activity Index on biopsy (NIH) (n = 80)	1 (IQR: 0–5)
Chronicity Index on biopsy (NIH) (n = 85)	3 (IQR: 1–4)
EXT1/EXT2 positivity (n = 97)	34/97 (35.1%)

Values are expressed as median (IQR) or n (%). IQR: Interquartile Range; SLE: Systemic Lupus Erythematosus; ANA: Antinuclear Antibody; RAAS: Renin–Angiotensin–Aldosterone System; LMN: Lupus Membranous Nephritis; EXT: Exostosin.

**Table 2:** Clinical characteristics of EXT1-positive and EXT1-negative patients on diagnosis.

Characteristics	EXT1-Positive (n = 34)	EXT1-Negative (n = 63)	*p* Value
Age (years)	37 (IQR: 30–42)	34 (IQR: 27–41)	0.427
Female sex	27 (79.4%)	57 (90.5%)	0.127
Race/ethnicity (n = 96)	1/33 (3.0%) Asian/8/33 (24.2%) Black or mixed-race/ 24/33 (72.7%) White	0/63 Asian/25/63 (39.6%) Black or mixed-race 38/63 (60.3%) White	0.268
Prior SLE diagnosis (n = 94)	22/32 (68.8%)	49/63 (79.0%)	0.271
Positive ANA (n = 67)	22/25 (88.0%)	41/42 (97.6%)	0.108
Diagnostic Criteria			
Constitutional symptoms (n = 94)	0/32	3/62 (4.8%)	0.206
Hematological (n = 94)	7/32 (21.9%)	19/62 (30.6%)	0.367
Neuropsychiatric (n = 94)	3/32 (9.4%)	3/62 (4.8%)	0.393
Mucocutaneous (n = 94)	13/32 (40.6%)	22/62 (35.5%)	0.625
Serositis (n = 94)	5/32 (15.6%)	10/62 (16.1%)	0.949
Joint involvement (n = 94)	12/31 (38.7%)	29/62 (46.8%)	0.460
C3 consumption (n = 97)	13 (38.2%)	19 (20.6%)	0.419
C4 consumption (n = 97)	11 (30.6%)	15 (24.6%)	0.061
Antiphospholipid antibodies (n = 59)	8/20 (40.0%)	6/39 (15.4%)	**0.035**
Anti-dsDNA or anti-Sm positive (n = 92)	22/31 (71.0%)	42/61 (68.9%)	0.834
Induction therapy (n = 92)	9/32 (28.1%)	22/60 (36.7%)	0.409
Prior induction treatment duration (months)	5 (IQR: 3–7.5)	4 (IQR: 2,5–6)	0.671
Cyclophosphamide	5/32 (15.6%)	15/60 (25.0%)	0.299
Mycophenolate	4/32 (12.5%)	6/60 (10.0%)	0.713
Rituximab	0	1/60 (1.7%)	0.462
Maintenance therapy (n = 92)	11/32 (34.4%)	24/60 (40.0%)	0.409
Azathioprine	4/32 (12.5%)	8/60 (13.3%)	0.910
Mycophenolate	6/32 (18.8%)	13/60 (21.7%)	0.742
Other	2/32 (6.3%)	2/60 (13.3%)	0.513
Hydroxychloroquine (n = 92)	17/32 (53.1%)	32/60 (53.3%)	0.948
Corticosteroids (n = 92)	24/32 (75.5%)	43/60 (71.7%)	0.732
RAAS blockers (n = 92)	20/32 (62.5%)	36/60 (60.0%)	0.815

Values are expressed as median (IQR) or n (%). IQR: Interquartile Range; SLE: Systemic Lupus Erythematosus; ANA: Antinuclear Antibody; RAAS: Renin–Angiotensin–Aldosterone System; EXT: Exostosin. Bold values indicate statistically significant results (*p* < 0.05).

**Table 3:** Laboratory and pathological characteristics of EXT1-positive and EXT1-negative patients on diagnosis.

Characteristics	EXT1-Positive (n = 34)	EXT1-Negative (n = 63)	*p* Value
Creatinine (mg/dL)	0.78 (IQR: 0.62–1.26)	0.83 (IQR: 0.64–1.41)	0.726
CKD-EPI 2021 eGFR (mL/min/1.73 m^2^)	104.5 (IQR: 73–120)	99 (IQR: 55–120)	0.426
Urine red blood cells (cells/field)	3 (IQR: 1–13)	6 (IQR: 2–16)	0.426
Proteinuria (g) (n = 96)	1.19 (IQR: 0.56–2.52)	2.03 (IQR: 0.84–4.48)	0.258
Nephrotic-range proteinuria (n = 96)	6/33 (18.2%)	20/63 (31.7%)	0.134
Serum albumin (g/dL) (n = 97)	3.3 (IQR 2.9–3.8)	3.2 (IQR: 2.4–3.6)	0.160
Serum total cholesterol (mg/dL) (n = 97)	232 (IQR: 195–254)	229 (IQR: 187–275)	0.716
C3 consumption (n = 97)	13/34 (38.2%)	19/63 (30.2%)	0.134
C4 consumption (n = 97)	13/34 (38.2%)	15/63 (23.8%)	0.958
Anti-dsDNA positive (n = 91)	20/30 (66.6%)	41/61 (67.2%)	0.419
Lupus membranous nephritis with proliferative component	21/34 (61.8%)	44/63 (69.8%)	0.908
Crescents on renal biopsy (n = 97)	9/34 (26.5%)	16/63 (15.4%)	0.908
C1q deposits (n = 93)	29/33 (87.5%)	53/60 (88.3%)	0.670
Activity Index on biopsy (NIH) (n = 80)	1 (IQR: 0–5) (n = 26)	2 (IQR: 0–4) (n = 54)	0.567
Chronicity Index on biopsy (NIH) (n = 85)	2.5 (IQR: 1–4) (n = 31)	3 (IQR: 1.5–4) (n = 54)	0.504

Values are expressed as median (IQR) or n (%). IQR: Interquartile Range; EXT: Exostosin.

**Table 4:** Clinical characteristics and outcomes in LMN patients after 1 year follow-up: EXT1-positive vs. EXT1-negative groups.

Characteristics After 1 Year	EXT1-Positive (n = 29)	EXT1-Negative (n = 59)	*p* Value
LMN with proliferative component	19/29 (65.5%)	43/59 (72.9%)	0.476
C3 consumption	8/26 (30.8%)	7/58 (12.1%)	**0.038**
C4 consumption	8/26 (30.8%)	6/58 (10.3%)	**0.020**
Anti-dsDNA positive	14/24 (58.3%)	33/55 (60.0%)	0.088
Proteinuria (g)	0.42 (IQR: 0.16–1.18)	0.46 (IQR: 0.15–1.08)	0.619
Nephrotic-range proteinuria	1/26 (4.0%)	5/59 (8.0%)	0.279
Creatinine (mg/dL)	0.86 (IQR: 0.59–1.29) (n = 27)	0.73 (IQR: 0.63–0.97) (n = 59)	0.488
CKD-EPI 2021 eGFR (mL/min/1.73 m^2^)	103 (IQR: 56–121) (n = 27)	108 (IQR: 80–120) (n = 59)	0.248
Complete Response	15/28 (54.0%)	26/59 (44.1%)	0.429
Primary Effective	2/28 (7.0%)	7/59 (11.9%)	
Partial Response	2/28 (7.0%)	11/59 (18.6%)	
No Response	9/28 (32.0%)	15/59 (25.4%)	
eGFR decline ≥40%	1/27 (3.7%)	1/59 (1.7%)	0.566
ESKD	1/28 (3.6%)	0/59	0.144
RRT	1/28 (3.6%)	0/59	0.144
Death	1/29 (3.4%)	0/59	0.151

Values are expressed as median (IQR) or n (%). EXT: Exostosin; IQR: Interquartile Range; eGFR: estimated Glomerular Filtration Rate; ESKD: End-Stage Kidney Disease; RRT: Renal Replacement Therapy; CNI: Calcineurin Inhibitors. Bold values indicate statistically significant results (*p* < 0.05).

**Table 5:** Clinical characteristics and outcomes in LMN patients after 2 years follow-up: EXT1-positive vs. EXT1-negative groups.

Characteristics After 2 Years	EXT1-Positive (n = 27)	EXT1-Negative (n = 57)	*p* Value
LMN with proliferative component	17/27 (63.0%)	41/57 (71.9%)	0.406
C3 consumption	4/27 (14.8%)	7/56 (12.5%)	0.770
C4 consumption	4/27 (14.8%)	6/56 (10.7%)	0.374
Anti-dsDNA positive	11/25 (44.0%)	27/50 (54.0%)	0.419
Proteinuria (g)	0.39 (IQR: 0.16–1.14) (n = 26)	0.39 (IQR: 0.15–0.81) (n = 56)	1
Nephrotic-range proteinuria	3/26 (7.7%)	3/56 (5.4%)	0.680
Creatinine (mg/dL)	0.73 (IQR: 0.64–1.29) (n = 27)	0.85 (IQR: 0.66–1.1) (n = 56)	0.533
CKD-EPI 2021 eGFR (mL/min/1.73 m^2^)	105 (IQR: 66–120) (n = 27)	98.5 (IQR: 67.5–117) (n = 56)	0.583
Complete Response	13/26 (50.0%)	29/56 (51.8%)	0.974
Primary Effective	3/26 (11.5%)	8/56 (14.3%)	
Partial Response	2/26 (7.7%)	4/56 (7.1%)	
No Response	8/26 (30.8%)	15/56 (26.8%)	
eGFR decline ≥40%	0/27	6/56 (10.5%)	0.077
ESKD	0/27	0/56	-
RRT	0/27	0/56	-
Death	0/27	1/57 (1.8%)	0.488

Values are expressed as median (IQR) or n (%). EXT: Exostosin; IQR: Interquartile Range; eGFR: estimated Glomerular Filtration Rate; ESKD: End-Stage Kidney Disease; RRT: Renal Replacement Therapy; CNI: Calcineurin Inhibitors.

**Table 6:** Clinical characteristics and outcomes in LMN patients after 3 years follow-up: EXT1-positive vs. EXT1-negative groups.

Characteristics After 3 Years	EXT1-Positive (n = 23)	EXT1-Negative (n = 49)	*p* Value
LMN with proliferative component	13/23 (56.5%)	36/49 (73.5%)	0.150
C3 consumption	1/23 (4.3%)	1/46 (2.2%)	0.611
C4 consumption	2/23 (8.7%)	4/46 (8.7%)	1
Anti-dsDNA positive	11/23 (47.8%)	28/45 (62.2%)	0.256
Proteinuria (g)	0.35 (IQR: 0.08–1.52) (n = 23)	0.35 (IQR: 0.13–0.76) (n = 45)	1
Nephrotic-range proteinuria	1/23 (4.3%)	2/45 (4.4%)	0.985
Creatinine (mg/dL)	0.82 (IQR: 0.64–1.33) (n = 23)	0.84 (IQR: 0.69–1.07) (n = 47)	0.800
CKD-EPI 2021 eGFR (mL/min/1.73 m^2^)	97 (IQR: 60–120) (n = 23)	99 (IQR: 65–118) (n = 47)	0.583
Complete Response	11/23 (47.8%)	22/45 (48.9%)	0.569
Primary Effective	2/23 (8.7%)	9/45 (20.0%)	
Partial Response	2/23 (8.7%)	2/45 (4.4%)	
No Response	8/23 (34.8%)	12/45 (26.7%)	
eGFR decline ≥40%	3/23 (13.0%)	3/57 (6.4%)	0.349
ESKD	0/23	1/49	0.481
RRT	0/23	0/49	-
Death	0/23	0/49	-

Values are expressed as median (IQR) or n (%). EXT: Exostosin; IQR: Interquartile Range; eGFR: estimated Glomerular Filtration Rate; ESKD: End-Stage Kidney Disease; RRT: Renal Replacement Therapy; CNI: Calcineurin Inhibitors.

**Table 7:** Clinical characteristics and outcomes in LMN patients after 4 years follow up: EXT1-positive vs. EXT1-negative groups.

Characteristics After 4 Years	EXT1-Positive (n = 16)	EXT1-Negative (n = 36)	*p* Value
LMN with proliferative component	8/16 (50.0%)	26/36 (72.2%)	0.150
C3 consumption	0/16	2/34 (2.9%)	0.322
C4 consumption	4/16 (25.0%)	2/34 (2.9%)	0.052
Anti-dsDNA positive	7/14 (50.0%)	21/30 (70.0%)	0.199
Proteinuria (g)	0.46 (IQR: 0.12–1.42) (n = 15)	0.27 (IQR: 0.13–0.72) (n = 34)	0.162
Nephrotic-range proteinuria	2/15 (13.3%)	1/34 (2.9%)	0.162
Creatinine (mg/dL)	0.81 (IQR: 0.65–1.17) (n = 16)	0.89 (IQR: 0.72–1.08) (n = 34)	1
CKD-EPI 2021 eGFR (mL/min/1.73 m^2^)	95 (IQR: 63–120) (n = 16)	91.5 (IQR: 72–114) (n = 34)	1
Complete Response	6/15 (40.0%)	16/36 (44.4%)	0.640
Primary Effective	3/15 (20.0%)	5/36 (13.9%)	
Partial Response	0/15	3/36 (8,3%)	
No Response	6/15 (40.0%)	12/36 (33.3%)	
eGFR decline ≥40%	2/16 (12.5%)	4/34 (11.8%)	0.940
ESKD	0/16	3/36 (8.3%)	0.234
RRT	0/16	2/36 (5.6%)	0.320
Death	0/16	0/36	-

Values are expressed as median (IQR) or n (%). EXT: Exostosin; IQR: Interquartile Range; eGFR: estimated Glomerular Filtration Rate; ESKD: End-Stage Kidney Disease; RRT: Renal Replacement Therapy; CNI: Calcineurin Inhibitors.

## Data Availability

The data presented in this study are available on request from the corresponding author due to institutional privacy regulations and patient confidentiality requirements.

## Supplementary Materials

The following supporting information can be downloaded at: https://www.mdpi.com/article/10.3390/diagnostics16111591/s1, Section S1: Immunohistochemical Analysis; Table S1: EXT1-Positive Patients Excluding Those on Induction Therapy at the Time of Biopsy; Table S2: Sample Size Evolution; Figure S1: Survival curve for ESKD; Figure S2: Survival curve for RRT; Figure S3: Evolution of creatinine between groups during follow-up; Figure S4: Evolution of proteinuria between groups during follow-up; Figure S5: Evolution of treatment responses over time adjusted for creatinine, proteinuria, activity and chronicity indices and C3 and C4 levels; Figure S6: Evolution of treatment responses over time adjusted for initial induction therapy used.

## Abbreviations

The following abbreviations are used in this manuscript:

ANA	Antinuclear Antibodies
CKD	Chronic Kidney Disease
CKD-EPI	Chronic Kidney Disease Epidemiology Collaboration
CNI	Calcineurin Inhibitor
ESKD	End-Stage Kidney Disease
EXT1	Exostosin 1
EXT2	Exostosin 2
GMB	Glomerular Basement Membrane
HS	Heparan Sulfate
IHQ	Immunohistochemistry
IQR	Interquartile Range
KDIGO	Kidney Disease: Improving Global Outcomes
LMN	Lupus Membranous Nephropathy
LN	Lupus Nephritis
MN	Membranous Nephropathy
NIH	National Institutes of Health (Activity Index)
PLA2R	M-type Phospholipase A2 Receptor
RAAS	Renin–Angiotensin–Aldosterone System
RRT	Renal Replacement Therapy SLE: Systemic Lupus Erythematosus
SLE	Systemic Lupus Erythematosus
THSD7A	Thrombospondin Type-1 Domain-Containing 7A
