# Supplementary material for: Detection of Exostosin 1 in Lupus Nephritis: Prevalence, Clinico–Pathologic and Renal Outcome Correlations
Source: Diagnostics (Basel). 2026 May 23;16(11):1591. doi: 10.3390/diagnostics16111591 (PMC13256564; doi:10.3390/diagnostics16111591)
Supplement: Supplementary file 1 [file diagnostics-16-01591-s001.zip › diagnostics-4285236-supplementary.pdf]

Detection of Exostosin 1 in Lupus Nephritis: Prevalence, Clinico-Pathologic and Renal Outcome Correlations

Supplementary Materials

S1. Immunohistochemical Analysis

Slides for immunohistochemical (IHC) analysis were obtained from paraffin blocks stored at the Department of Pathology of Hospital das Clínicas, corresponding to the selected patients. Tissue sectioning and IHC analysis were performed at the Histocell Solucoes em Anatomia Patologica laboratory.

Histological sections 3–4 μm thick were initially obtained. Slides were placed in an oven at 65°C for 40 minutes to remove excess paraffin, then subjected to the following procedures:

Rehydration in two baths of 99% ethanol, two baths of 96% ethanol, and one bath of 70% ethanol;

Washing in running water followed by distilled water;

Endogenous peroxidase blocking with 3% hydrogen peroxide for 10 minutes;

Washing in running water followed by distilled water;

Antigen retrieval in an electric pressure cooker for 15 minutes using ethylenediaminetetraacetic acid (EDTA) buffer pH 9.0 (Agilent);

Washing in running water followed by distilled water;

Three washes in phosphate-buffered saline (PBS) pH 7.2;

For EXT1, overnight incubation with a rabbit-derived anti-exostosin 1 polyclonal antibody at a 1:100 dilution (Thermo Scientific, #PA5-60699) in a humidified chamber;

Three washes in PBS pH 7.2;

Incubation with horseradish peroxidase (HRP) Polymer for 30 minutes in a humidified chamber;

Three washes in PBS pH 7.2;

Chromogenic detection with liquid 3,3'-diaminobenzidine (1 drop of diaminobenzidine + 1 mL of substrate);

Washing in running water followed by distilled water;

Counterstaining with Harris hematoxylin for 2 minutes;

Washing in running water followed by distilled water;

Dehydration in graded ethanol series (70%, 96%, 99%) followed by xylene;

Coverslipping with Entellan resin with sample identification.

Table S1. Clinical and Pathological Characteristics of EXT1-Positive and EXT1-Negative Patients at Diagnosis, Excluding Patients Already on Induction Therapy.

Characteristics	EXT1-Positive (n=25)	EXT1-Negative (n=41)	p Value
Age (years)	40 (IQR: 30-42)	35 (IQR: 26-41)	0.450
Female sex	22 (88.0%)	38 (92.7%)	0.520
Race/ethnicity (n = 65)	1/24 (4.2%) Asian / 5/24 (20.8%) Black or Mixed-race/ 18/24 (75.0%) White	0/41 Asian/ 19/41 (46.5%) Black or Mixed-race/ 22/41 (53.7%) White	0.133
Prior SLE diagnosis (n = 63)	13/23 (56.5%)	30/40 (75.0%)	0.129
Positive ANA (n=67)	19/22 (90.5%)	27/28 (96.4%)	0.389
Diagnostic Criteria			
Constitutional symptoms (n = 63)	0/23	2/40 (5.0%)	0.275
Hematological (n = 63)	3/23 (13.0%)	13/40 (32.5%)	0.087
Neuropsychiatric (n = 63	2/23 (8.6%)	1/40 (2.5%)	0.266
Mucocutaneous (n = 63)	8/23 (34.8%)	13/40 (12.5%)	0.853
Serositis (n = 63)	4/23 (17.4%)	5/40 (12.5%)	0.593
Joint involvement (n = 62)	7/22 (31.8%)	15/40 (37.5%)	0.654
C3 consumption (n = 66	11/25 (44.0%)	13/41 (31.7%)	0.313
C4 consumption (n = 66)	9/25 (36.0%)	10/41 (24.4%)	0.312
Antiphospholipid antibodies (n = 38)	7/15 (46.7%)	4/23 (17.4%)	0.051
Anti-dsDNA or anti-Sm positive (n = 62	16/23 (69.6%)	29/39 (74.4%)	0.682
Maintenance therapy (n = 61)	7/23 (30.4%)	19/38 (50.0%)	0.134
Azathioprine	2/23 (8.6%)	8/38 (21.1%)	0.206
Mycophenolate	3/23 (13.0%)	9/38 (23.7%)	0.311
Other	2/23 (8.6%)	1/38 (2.6%)	0.288
Hydroxychloroquine (n = 61)	11/23 (47.8%)	18/38 (47.4%)	0.971
Corticosteroids (n = 61)	16/23 (65.5%)	22/38 (57.9%)	0.362
AAS blockers (n = 61)	11/23 (47.8%)	17/38 (44.7%)	0.814
Creatinine (mg/dL)	0.84 (IQR: 0.69-1.27)	0.81 (IQR: 0.64-1.45)	0.801
CKD-EPI 2021 eGFR (mL/min/1.73 m²)	102 (IQR: 63-112)	102 (IQR: 54-120)	0.931
Urine red blood cells (cells/field)	3 (IQR: 1-12)	6 (IQR: 2-24)	0.246
Proteinuria (g) (n = 65)	0.87 (IQR: 0.52-2.91)	2.37 (IQR: 1.2-4.48)	0.246
Nephrotic-range proteinuria (n = 65)	5/24 (20.8%)	14/41 (34.1%)	0.150
Serum albumin (g/dL) (n = 66)	3.4 (IQR 3.0-3.8)	3.1 (IQR: 2.3-3.6)	0.450
Serum total cholesterol (mg/dL) (n = 63)	233 (IQR: 172-256) (n=24)	229 (IQR: 179-276) (n=39)	0.921
C3 consumption (n = 66)	13/41 (32.0%)	11/25 (44.0%)	0.313
C4 consumption (n = 66)	9/25 (36.0%)	11/41 (27.0%)	0.431
Anti-dsDNA positive (n = 61)	13/41 (32.0%)	11/25 (44.0%)	0.313

Lupus membranous nephritis with proliferative component	16/25 (64.0%)	26/41 (63.4%)	0.961
Crescents on renal biopsy (n = 66)	7/25 (28.0%)	12/41 (29.0%)	0.961
C1q deposits (n = 65)	23/25 (92.0%)	36/40 (90.0%)	0.786
Activity Index on biopsy (NIH) (n = 53)	1,5<br>(IQR: 0-5) (n=20)	2<br>(IQR: 0-6)(n=33)	0.464
Chronicity Index on biopsy (NIH) (n = 57)	2.0<br>(IQR: 1-4)(n=23)	3 (IQR: 1.5-<br>3.75)(n=34)	0.381

Values are expressed as median (IQR) or n (%). IQR: Interquartile Range; SLE: Systemic Lupus Erythematosus; ANA: Antinuclear Antibody; RAAS: Renin–Angiotensin–Aldosterone System; EXT: Exostosin.

Table S2. Sample Size Evolution.

	**EXT1-Positive**	**EXT1-Negative**
On Diagnosis	n=34<br>Data unavailability: 5	n=63<br>Data unavailability: 9
After 1 year	n=29<br>Death:1<br>Loss to follow-up: 1	n=59<br>Loss to follow-up: 2
After 2 years	n=27<br>Censoring: 4	n=57<br>Death: 1<br>Censoring:7
After 3 years	n=23<br>Censoring: 7	n=49<br>Censoring:7
After 4 years	n=16<br>Censoring: 4	n=36<br>RRT: 2<br>Censoring: 16
After 5 years	n=12<br>Loss to follow-up: 1<br>Censoring: 1	n=18<br>RRT: 1<br>Censoring: 5
After 6 years	n=10<br>Censoring: 2	n=12<br>Censoring: 6
After 7 years	n=8	n=6

EXT: Exostosin; RRT: Renal Replacement Therapy.

Figure S1. Survival curve for ESKD

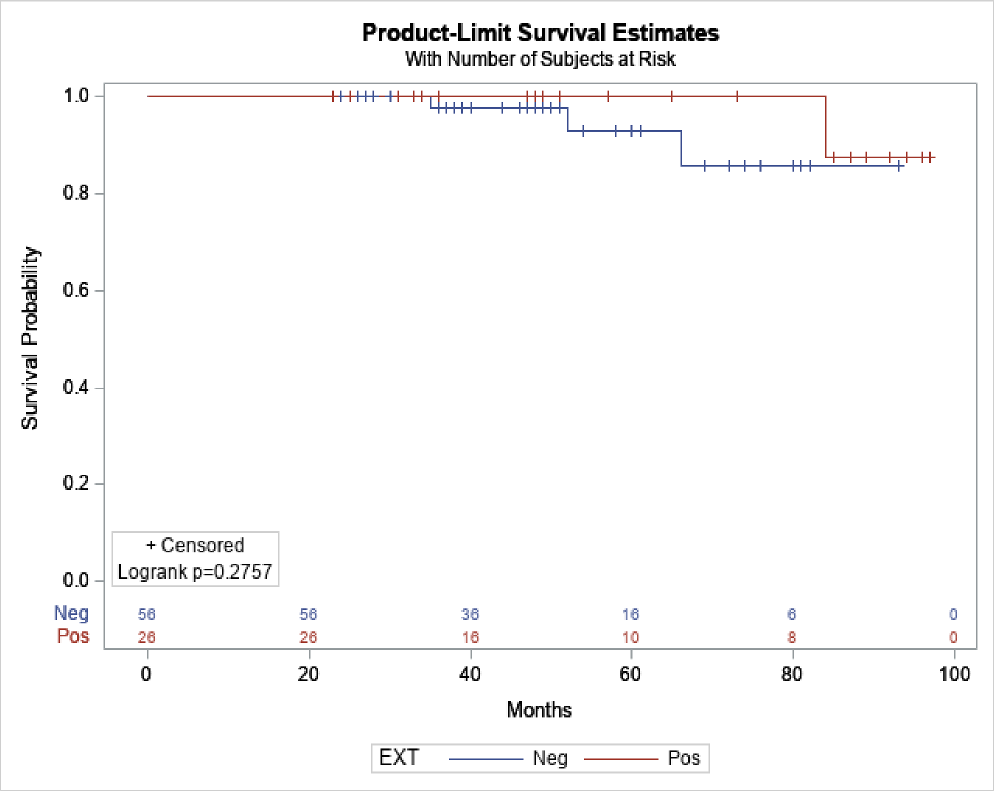

Figure S2. Survival curve for RRT

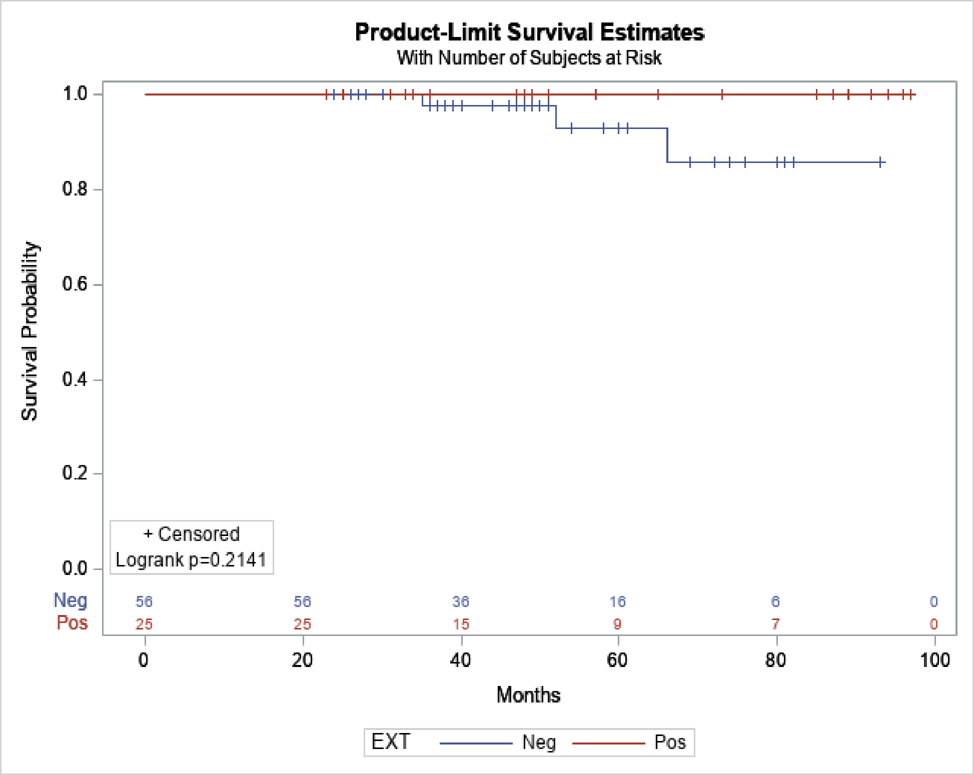

Figure S3. Evolution of creatinine between groups during follow-up.

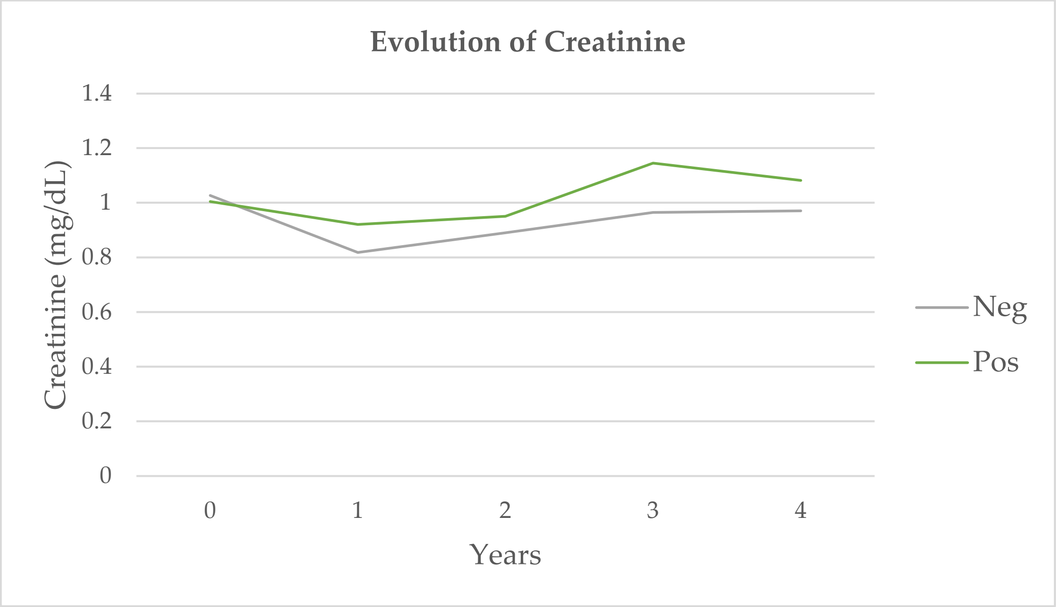

Adjusted for activity and chronicity indices and C3 and C4 levels.

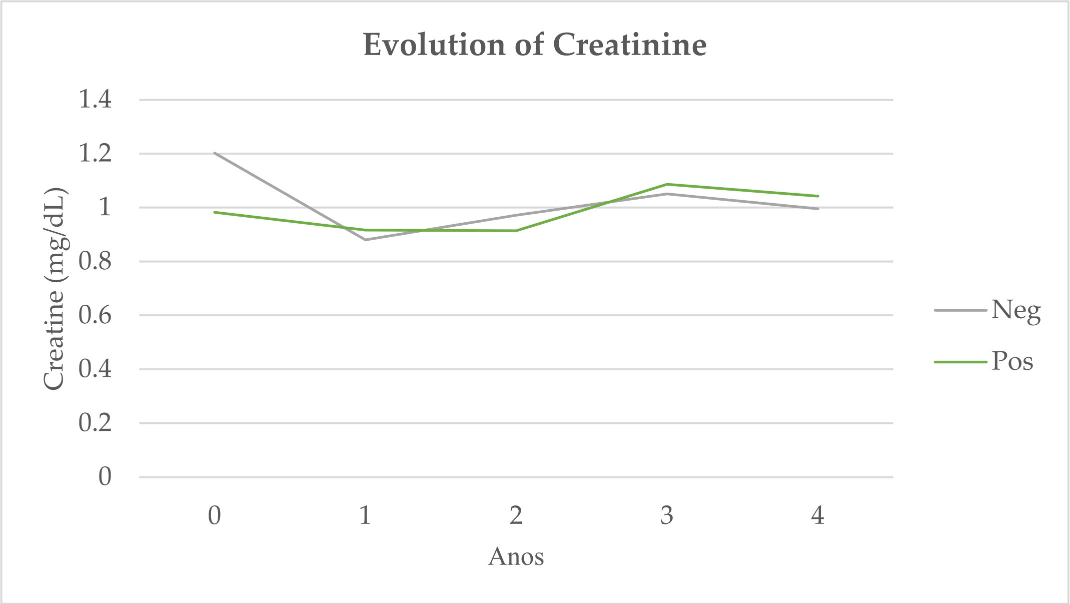

Adjusted for the initial induction therapy used.

Figure S4. Evolution of proteinuria between groups during follow-up.

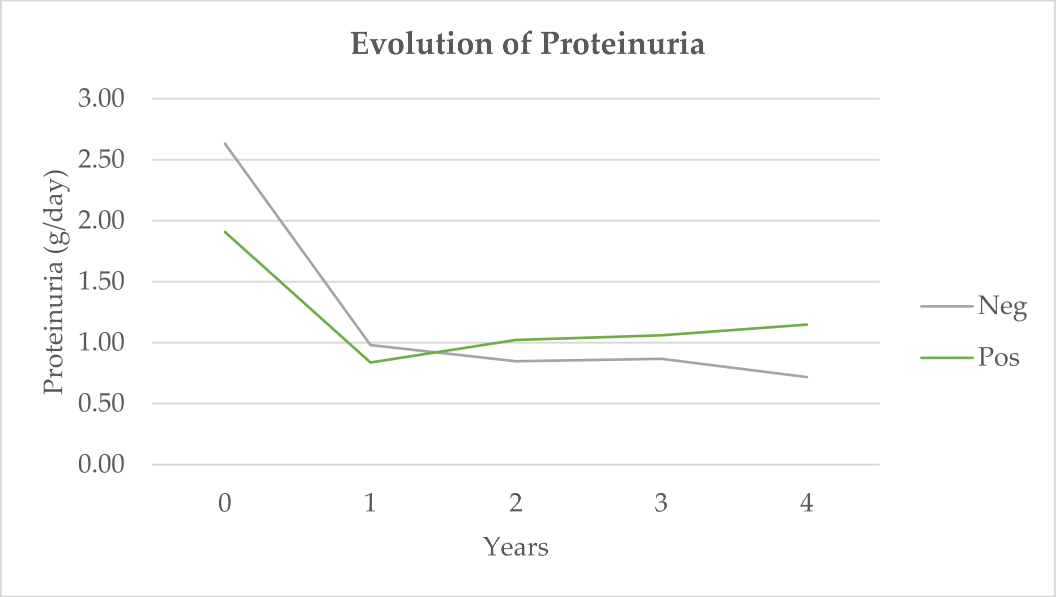

Adjusted for activity and chronicity indices and C3 and C4 levels.

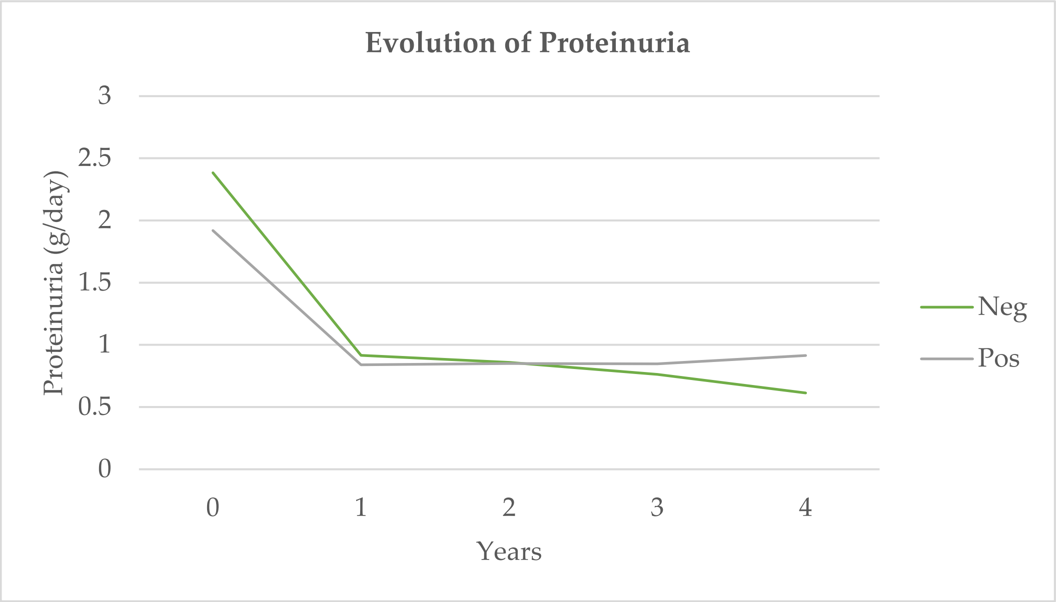

Adjusted for the initial induction therapy used.

Figure S5. Evolution of treatment responses over time adjusted for creatinine, proteinuria, activity and chronicity indices and C3 and C4 levels.

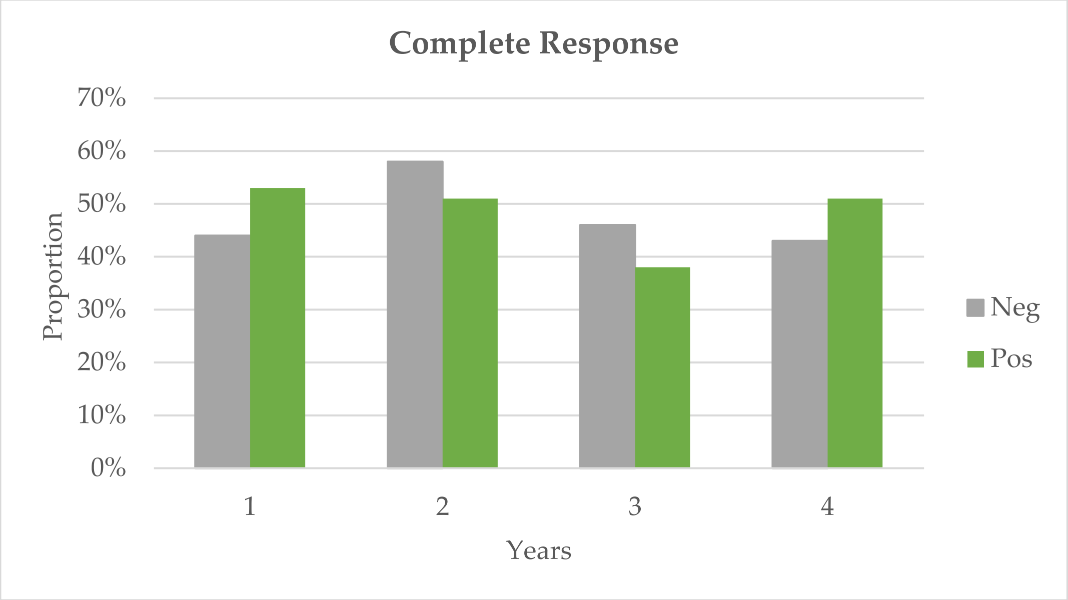

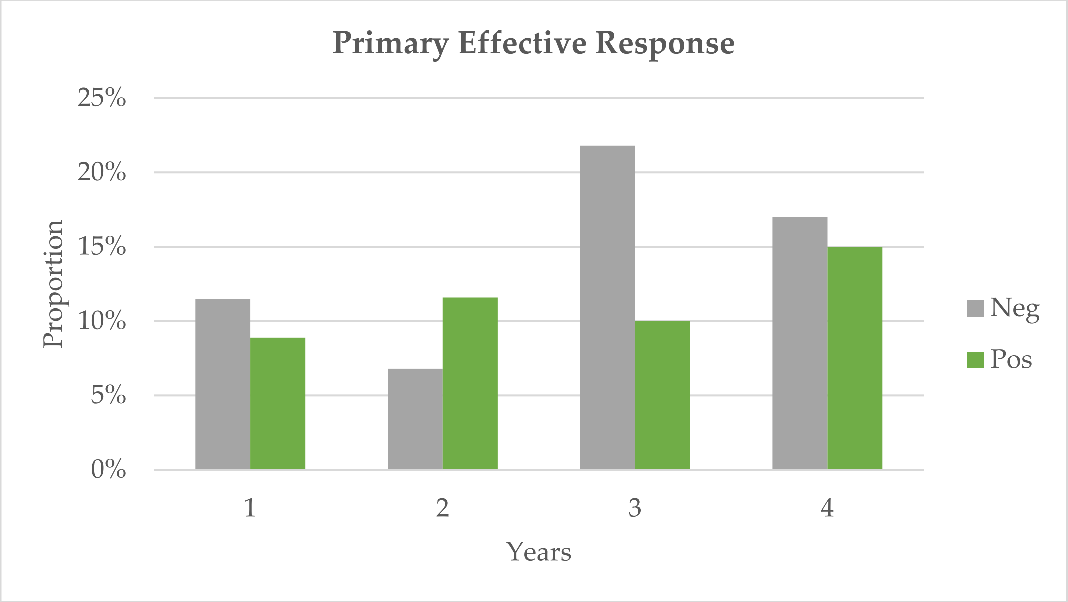

Continue Continuation

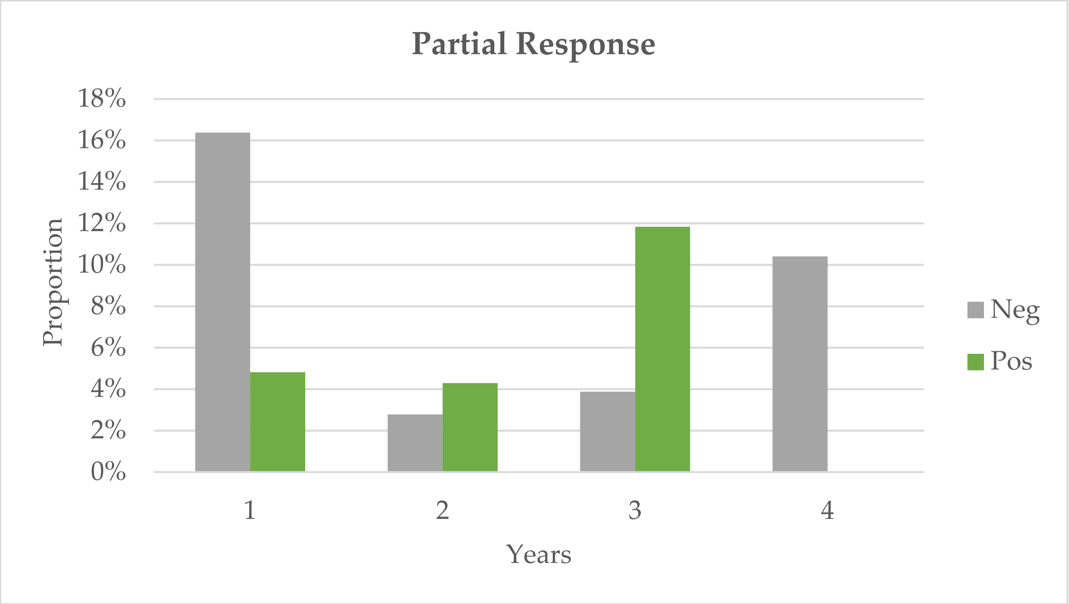
Partial Response
18%
16%
14%
12%
10%
8%
6%
4%
2%
0%
Proportion
1
2
3
4
Years
Neg
Pos

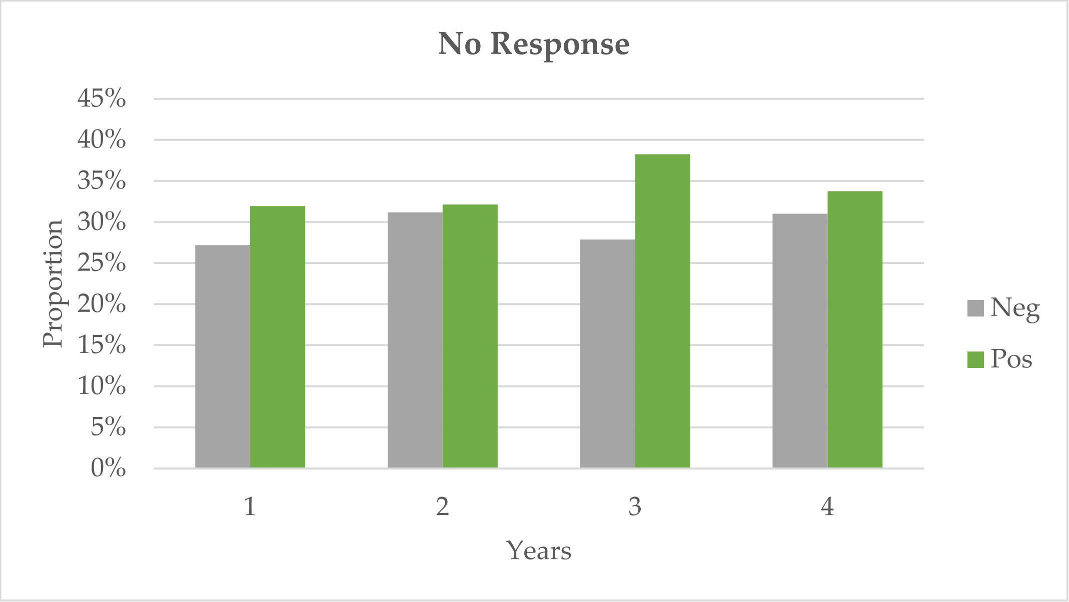
No Response
45%
40%
35%
30%
25%
20%
15%
10%
5%
0%
Proportion
1
2
3
4
Years
Neg
Pos

Figure S6. Evolution of treatment responses over time adjusted for initial induction therapy used.

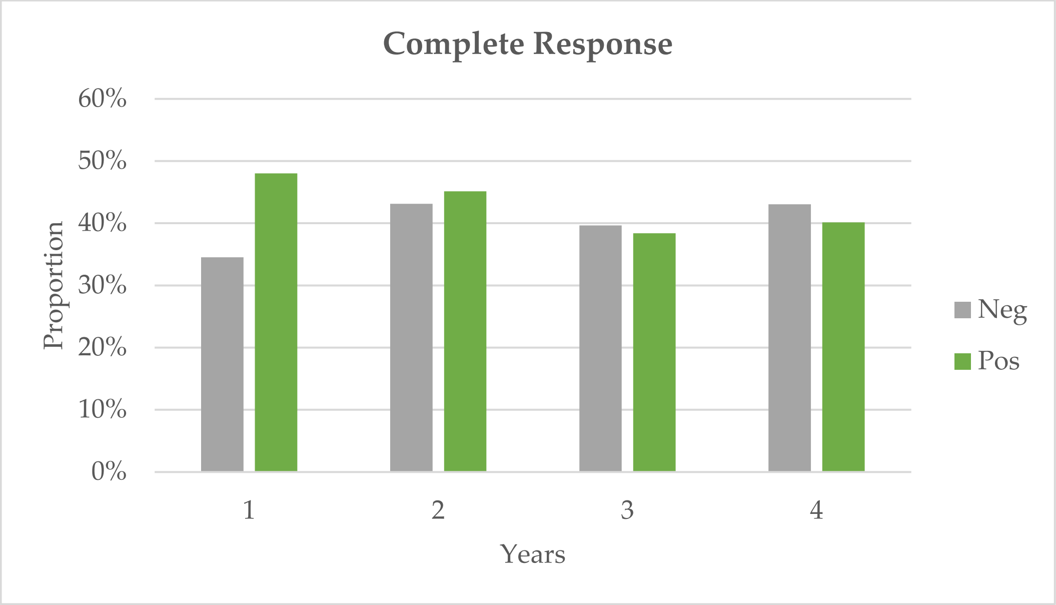

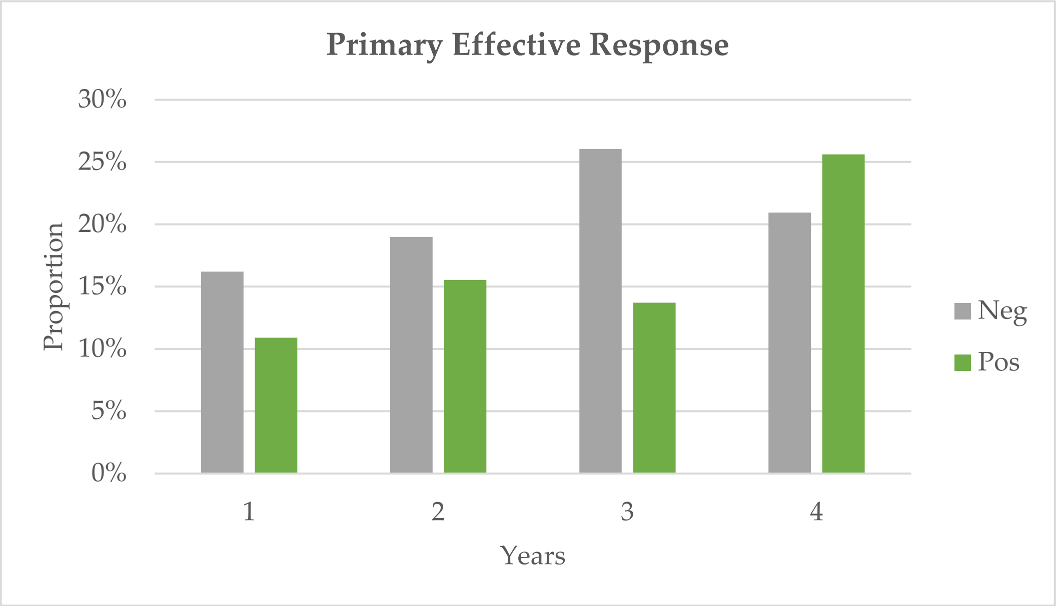

Continue Continuation

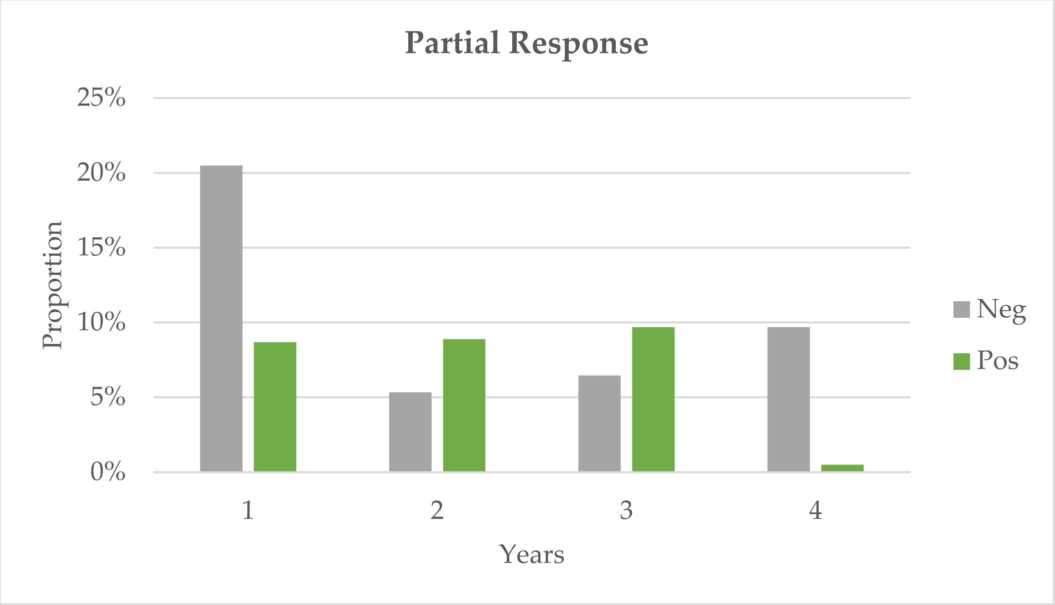
Partial Response
25%
20%
15%
10%
5%
0%
Proportion
1
2
3
4
Years
Neg
Pos

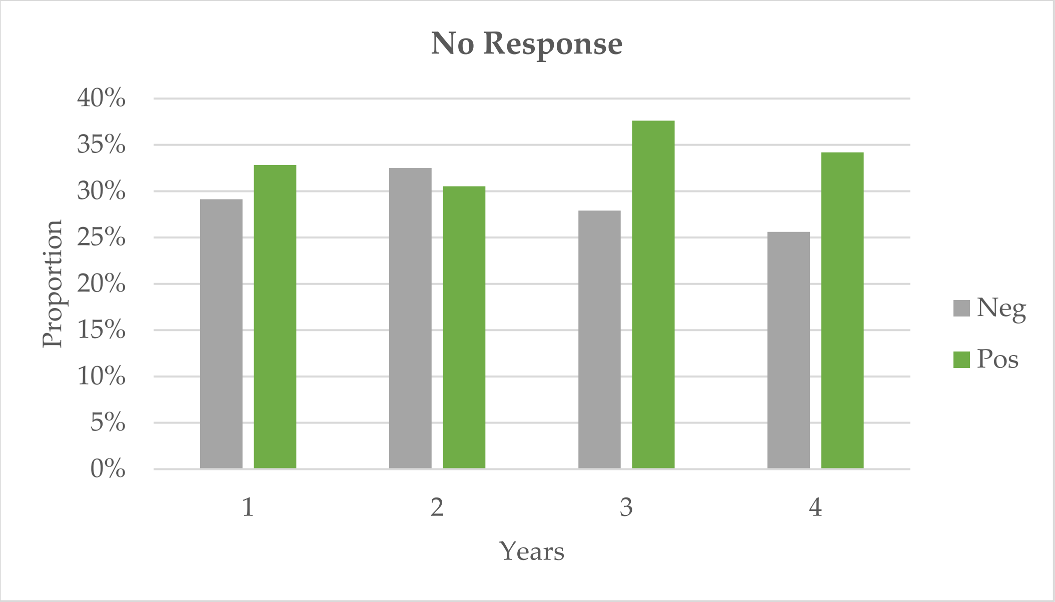
No Response
40%
35%
30%
25%
20%
15%
10%
5%
0%
Proportion
1
2
3
4
Years
Neg
Pos
